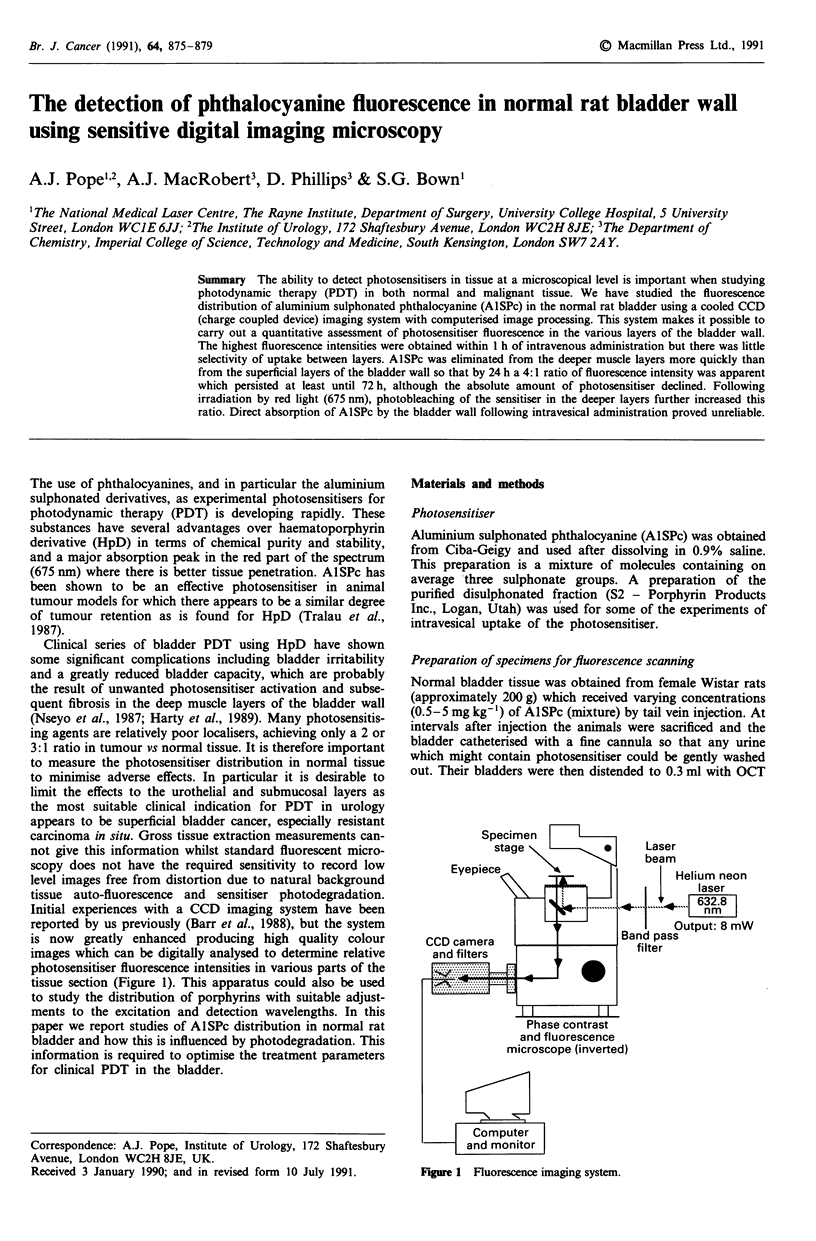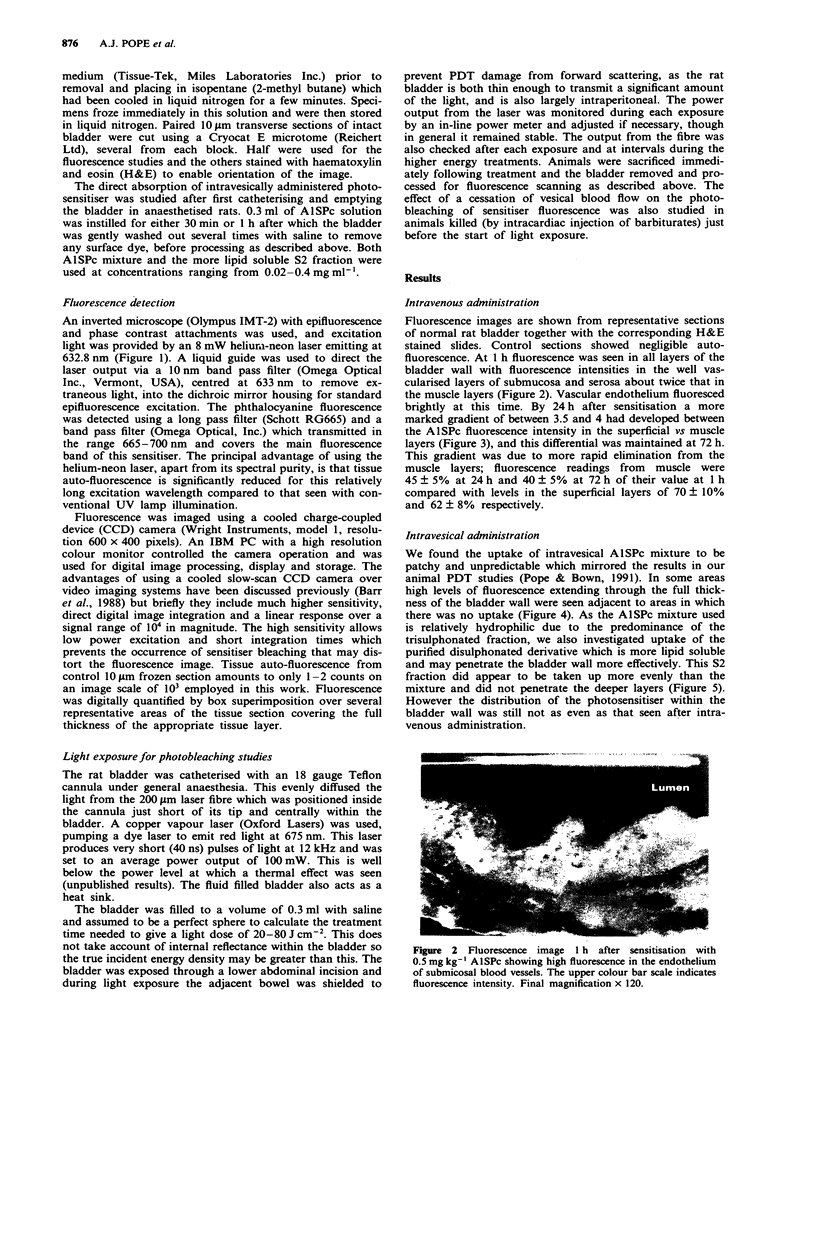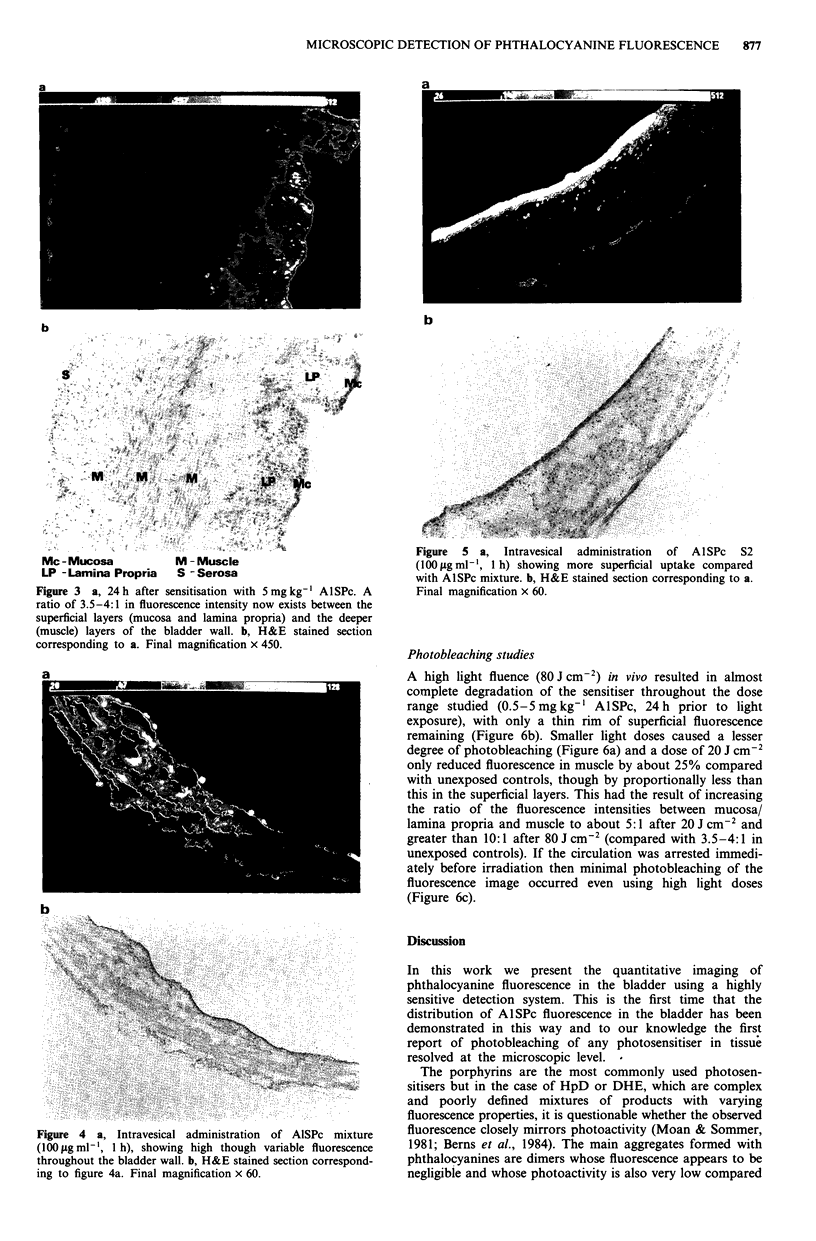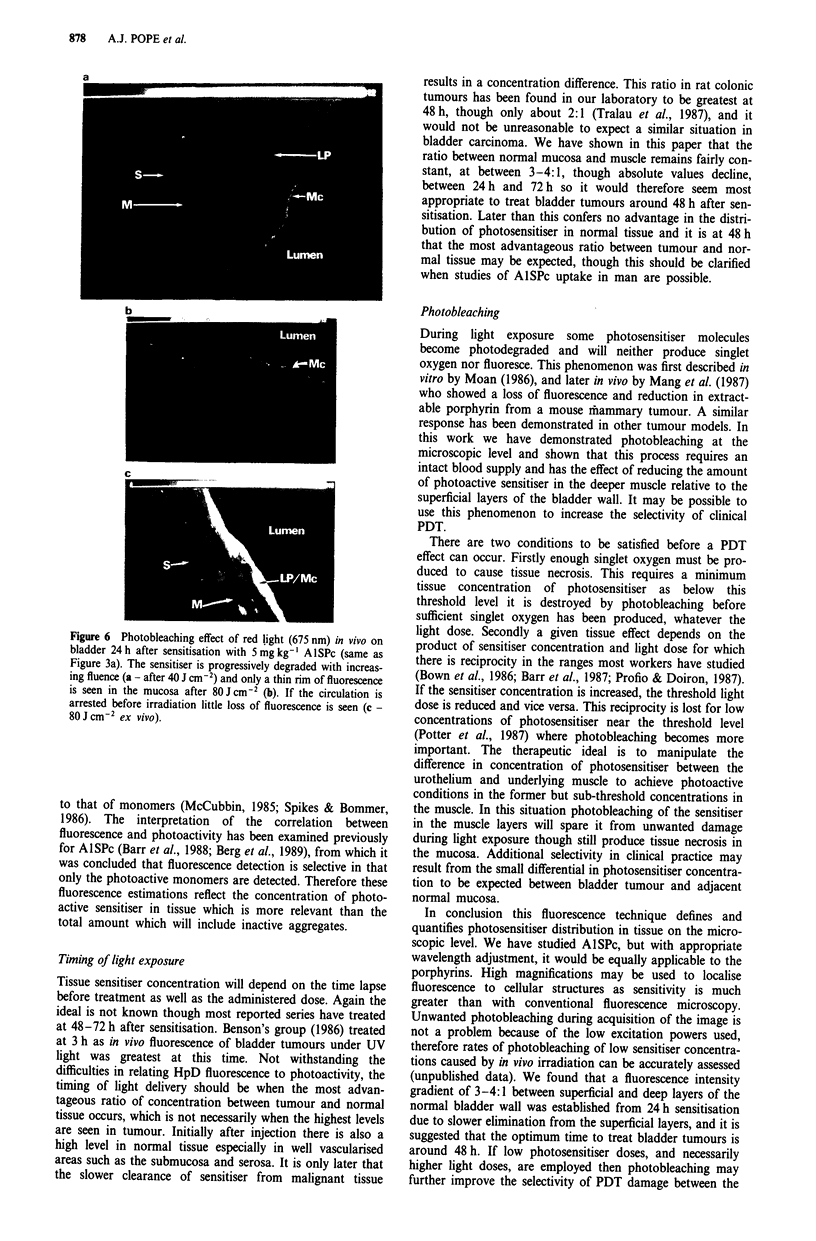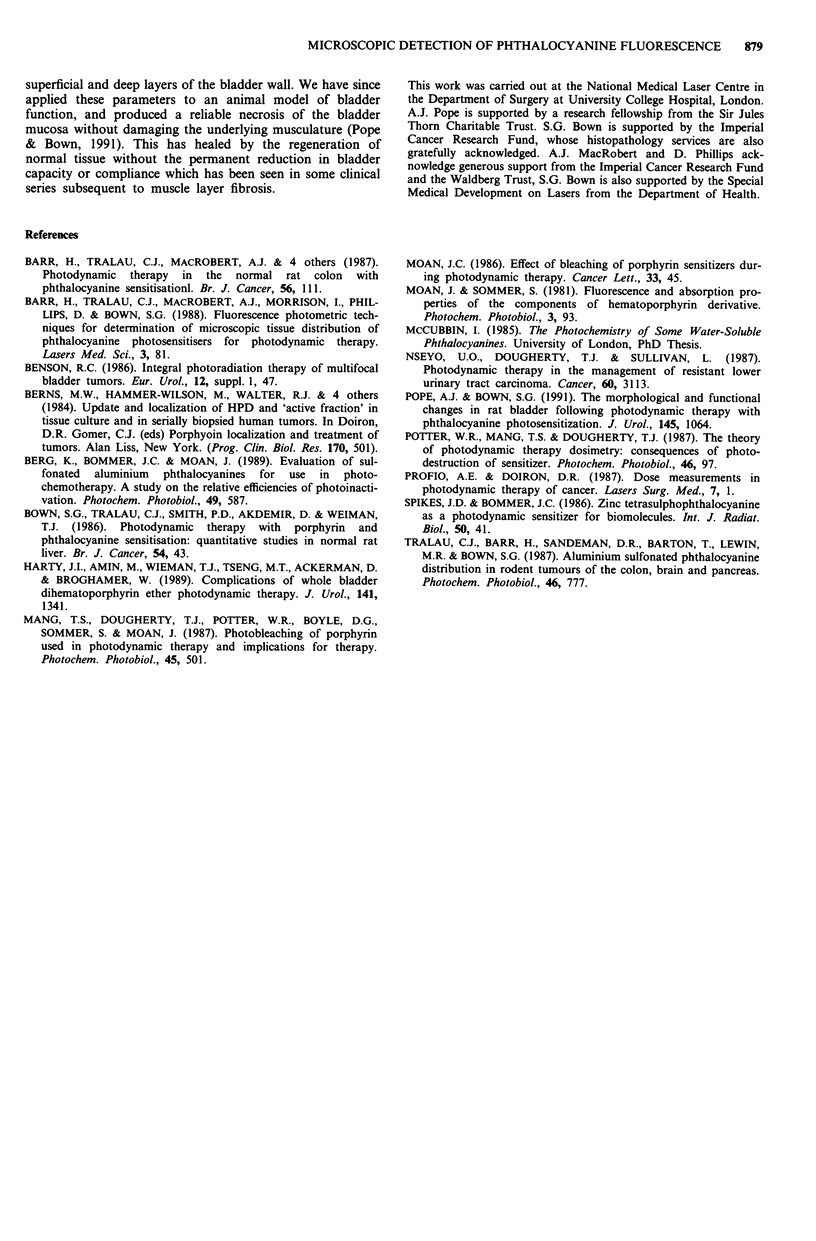# The detection of phthalocyanine fluorescence in normal rat bladder wall using sensitive digital imaging microscopy.

**DOI:** 10.1038/bjc.1991.417

**Published:** 1991-11

**Authors:** A. J. Pope, A. J. MacRobert, D. Phillips, S. G. Bown

**Affiliations:** National Medical Laser Centre, Rayne Institute, London, UK.

## Abstract

**Images:**


*Br. J. Cancer* (1991), **64**, 875–879 © Macmillan Press Ltd., 1991

### The detection of phthalocyanine fluorescence in normal rat bladder wall using sensitive digital imaging microscopy

A.J. Pope[1,2], A.J. MacRobert[3], D. Phillips[3] & S.G. Bown[1]

[1]*The National Medical Laser Centre, The Rayne Institute, Department of Surgery, University College Hospital, 5 University Street, London WC1E 6JJ;* [2]*The Institute of Urology, 172 Shaftesbury Avenue, London WC2H 8JE;* [3]*The Department of Chemistry, Imperial College of Science, Technology and Medicine, South Kensington, London SW7 2AY.*

**Summary** The ability to detect photosensitisers in tissue at a microscopical level is important when studying photodynamic therapy (PDT) in both normal and malignant tissue. We have studied the fluorescence distribution of aluminium sulphonated phthalocyanine (AlSPc) in the normal rat bladder using a cooled CCD (charge coupled device) imaging system with computerised image processing. This system makes it possible to carry out a quantitative assessment of photosensitiser fluorescence in the various layers of the bladder wall. The highest fluorescence intensities were obtained within 1 h of intravenous administration but there was little selectivity of uptake between layers. AlSPc was eliminated from the deeper muscle layers more quickly than from the superficial layers of the bladder wall so that by 24 h a 4:1 ratio of fluorescence intensity was apparent which persisted at least until 72 h, although the absolute amount of photosensitiser declined. Following irradiation by red light (675 nm), photobleaching of the sensitiser in the deeper layers further increased this ratio. Direct absorption of AlSPc by the bladder wall following intravesical administration proved unreliable.

The use of phthalocyanines, and in particular the aluminium sulphonated derivatives, as experimental photosensitisers for photodynamic therapy (PDT) is developing rapidly. These substances have several advantages over haematoporphyrin derivative (HpD) in terms of chemical purity and stability, and a major absorption peak in the red part of the spectrum (675 nm) where there is better tissue penetration. AlSPc has been shown to be an effective photosensitiser in animal tumour models for which there appears to be a similar degree of tumour retention as is found for HpD (Tralau *et al.*, 1987).

Clinical series of bladder PDT using HpD have shown some significant complications including bladder irritability and a greatly reduced bladder capacity, which are probably the result of unwanted photosensitiser activation and subsequent fibrosis in the deep muscle layers of the bladder wall (Nseyo *et al.*, 1987; Harty *et al.*, 1989). Many photosensitising agents are relatively poor localisers, achieving only a 2 or 3:1 ratio in tumour *vs* normal tissue. It is therefore important to measure the photosensitiser distribution in normal tissue to minimise adverse effects. In particular it is desirable to limit the effects to the urothelial and submucosal layers as the most suitable clinical indication for PDT in urology appears to be superficial bladder cancer, especially resistant carcinoma *in situ*. Gross tissue extraction measurements cannot give this information whilst standard fluorescent microscopy does not have the required sensitivity to record low level images free from distortion due to natural background tissue auto-fluorescence and sensitiser photodegradation. Initial experiences with a CCD imaging system have been reported by us previously (Barr *et al.*, 1988), but the system is now greatly enhanced producing high quality colour images which can be digitally analysed to determine relative photosensitiser fluorescence intensities in various parts of the tissue section (Figure 1). This apparatus could also be used to study the distribution of porphyrins with suitable adjustments to the excitation and detection wavelengths. In this paper we report studies of AlSPc distribution in normal rat bladder and how this is influenced by photodegradation. This information is required to optimise the treatment parameters for clinical PDT in the bladder.

Correspondence: A.J. Pope, Institute of Urology, 172 Shaftesbury Avenue, London WC2H 8JE, UK.
Received 3 January 1990; and in revised form 10 July 1991.

### Materials and methods

### *Photosensitiser*

Aluminium sulphonated phthalocyanine (AlSPc) was obtained from Ciba-Geigy and used after dissolving in 0.9% saline. This preparation is a mixture of molecules containing on average three sulphonate groups. A preparation of the purified disulphonated fraction (S2 – Porphyrin Products Inc., Logan, Utah) was used for some of the experiments of intravesical uptake of the photosensitiser.

### *Preparation of specimens for fluorescence scanning*

Normal bladder tissue was obtained from female Wistar rats (approximately 200 g) which received varying concentrations (0.5–5 mg $kg^{-1}$) of AlSPc (mixture) by tail vein injection. At intervals after injection the animals were sacrificed and the bladder catheterised with a fine cannula so that any urine which might contain photosensitiser could be gently washed out. Their bladders were then distended to 0.3 ml with OCT

**Figure 1** Fluorescence imaging system.

medium (Tissue-Tek, Miles Laboratories Inc.) prior to removal and placing in isopentane (2-methyl butane) which had been cooled in liquid nitrogen for a few minutes. Specimens froze immediately in this solution and were then stored in liquid nitrogen. Paired 10 µm transverse sections of intact bladder were cut using a Cryocat E microtome (Reichert Ltd), several from each block. Half were used for the fluorescence studies and the others stained with haematoxylin and eosin (H&E) to enable orientation of the image.

The direct absorption of intravesically administered photosensitiser was studied after first catheterising and emptying the bladder in anaesthetised rats. 0.3 ml of AlSPc solution was instilled for either 30 min or 1 h after which the bladder was gently washed out several times with saline to remove any surface dye, before processing as described above. Both AlSPc mixture and the more lipid soluble S2 fraction were used at concentrations ranging from 0.02–0.4 mg $ml^{-1}$.

### *Fluorescence detection*

An inverted microscope (Olympus IMT-2) with epifluorescence and phase contrast attachments was used, and excitation light was provided by an 8 mW helium-neon laser emitting at 632.8 nm (Figure 1). A liquid guide was used to direct the laser output via a 10 nm band pass filter (Omega Optical Inc., Vermont, USA), centred at 633 nm to remove extraneous light, into the dichroic mirror housing for standard epifluorescence excitation. The phthalocyanine fluorescence was detected using a long pass filter (Schott RG665) and a band pass filter (Omega Optical, Inc.) which transmitted in the range 665–700 nm and covers the main fluorescence band of this sensitiser. The principal advantage of using the helium-neon laser, apart from its spectral purity, is that tissue auto-fluorescence is significantly reduced for this relatively long excitation wavelength compared to that seen with conventional UV lamp illumination.

Fluorescence was imaged using a cooled charge-coupled device (CCD) camera (Wright Instruments, model 1, resolution 600 × 400 pixels). An IBM PC with a high resolution colour monitor controlled the camera operation and was used for digital image processing, display and storage. The advantages of using a cooled slow-scan CCD camera over video imaging systems have been discussed previously (Barr *et al.*, 1988) but briefly they include much higher sensitivity, direct digital image integration and a linear response over a signal range of $10^4$ in magnitude. The high sensitivity allows low power excitation and short integration times which prevents the occurrence of sensitiser bleaching that may distort the fluorescence image. Tissue auto-fluorescence from control 10 µm frozen section amounts to only 1–2 counts on an image scale of $10^3$ employed in this work. Fluorescence was digitally quantified by box superimposition over several representative areas of the tissue section covering the full thickness of the appropriate tissue layer.

### *Light exposure for photobleaching studies*

The rat bladder was catheterised with an 18 gauge Teflon cannula under general anaesthesia. This evenly diffused the light from the 200 µm laser fibre which was positioned inside the cannula just short of its tip and centrally within the bladder. A copper vapour laser (Oxford Lasers) was used, pumping a dye laser to emit red light at 675 nm. This laser produces very short (40 ns) pulses of light at 12 kHz and was set to an average power output of 100 mW. This is well below the power level at which a thermal effect was seen (unpublished results). The fluid filled bladder also acts as a heat sink.

The bladder was filled to a volume of 0.3 ml with saline and assumed to be a perfect sphere to calculate the treatment time needed to give a light dose of 20–80 J $cm^{-2}$. This does not take account of internal reflectance within the bladder so the true incident energy density may be greater than this. The bladder was exposed through a lower abdominal incision and during light exposure the adjacent bowel was shielded to prevent PDT damage from forward scattering, as the rat bladder is both thin enough to transmit a significant amount of the light, and is also largely intraperitoneal. The power output from the laser was monitored during each exposure by an in-line power meter and adjusted if necessary, though in general it remained stable. The output from the fibre was also checked after each exposure and at intervals during the higher energy treatments. Animals were sacrificed immediately following treatment and the bladder removed and processed for fluorescence scanning as described above. The effect of a cessation of vesical blood flow on the photobleaching of sensitiser fluorescence was also studied in animals killed (by intracardiac injection of barbiturates) just before the start of light exposure.

### Results

### *Intravenous administration*

Fluorescence images are shown from representative sections of normal rat bladder together with the corresponding H&E stained slides. Control sections showed negligible autofluorescence. At 1 h fluorescence was seen in all layers of the bladder wall with fluorescence intensities in the well vascularised layers of submucosa and serosa about twice that in the muscle layers (Figure 2). Vascular endothelium fluoresced brightly at this time. By 24 h after sensitisation a more marked gradient of between 3.5 and 4 had developed between the AlSPc fluorescence intensity in the superficial *vs* muscle layers (Figure 3), and this differential was maintained at 72 h. This gradient was due to more rapid elimination from the muscle layers; fluorescence readings from muscle were 45 ± 5% at 24 h and 40 ± 5% at 72 h of their value at 1 h compared with levels in the superficial layers of 70 ± 10% and 62 ± 8% respectively.

### *Intravesical administration*

We found the uptake of intravesical AlSPc mixture to be patchy and unpredictable which mirrored the results in our animal PDT studies (Pope & Bown, 1991). In some areas high levels of fluorescence extending through the full thickness of the bladder wall were seen adjacent to areas in which there was no uptake (Figure 4). As the AlSPc mixture used is relatively hydrophilic due to the predominance of the trisulphonated fraction, we also investigated uptake of the purified disulphonated derivative which is more lipid soluble and may penetrate the bladder wall more effectively. This S2 fraction did appear to be taken up more evenly than the mixture and did not penetrate the deeper layers (Figure 5). However the distribution of the photosensitiser within the bladder wall was still not as even as that seen after intravenous administration.

**Figure 2** Fluorescence image 1 h after sensitisation with 0.5 mg $kg^{-1}$ AlSPc showing high fluorescence in the endothelium of submicosal blood vessels. The upper colour bar scale indicates fluorescence intensity. Final magnification × 120.

**Figure 3** **a**, 24 h after sensitisation with 5 mg kg$^{-1}$ AlSPc. A ratio of 3.5–4:1 in fluorescence intensity now exists between the superficial layers (mucosa and lamina propria) and the deeper (muscle) layers of the bladder wall. **b**, H&E stained section corresponding to **a**. Final magnification × 450.

**Figure 4** **a**, Intravesical administration of AlSPc mixture (100 μg ml$^{-1}$, 1 h), showing high though variable fluorescence throughout the bladder wall. **b**, H&E stained section corresponding to figure 4a. Final magnification × 60.

**Figure 5** **a**, Intravesical administration of AlSPc S2 (100 μg ml$^{-1}$, 1 h) showing more superficial uptake compared with AlSPc mixture. **b**, H&E stained section corresponding to **a**. Final magnification × 60.

*Photobleaching studies*

A high light fluence (80 J cm$^{-2}$) *in vivo* resulted in almost complete degradation of the sensitiser throughout the dose range studied (0.5–5 mg kg$^{-1}$ AlSPc, 24 h prior to light exposure), with only a thin rim of superficial fluorescence remaining (Figure 6b). Smaller light doses caused a lesser degree of photobleaching (Figure 6a) and a dose of 20 J cm$^{-2}$ only reduced fluorescence in muscle by about 25% compared with unexposed controls, though by proportionally less than this in the superficial layers. This had the result of increasing the ratio of the fluorescence intensities between mucosa/lamina propria and muscle to about 5:1 after 20 J cm$^{-2}$ and greater than 10:1 after 80 J cm$^{-2}$ (compared with 3.5–4:1 in unexposed controls). If the circulation was arrested immediately before irradiation then minimal photobleaching of the fluorescence image occurred even using high light doses (Figure 6c).

### Discussion

In this work we present the quantitative imaging of phthalocyanine fluorescence in the bladder using a highly sensitive detection system. This is the first time that the distribution of AlSPc fluorescence in the bladder has been demonstrated in this way and to our knowledge the first report of photobleaching of any photosensitiser in tissue resolved at the microscopic level.

The porphyrins are the most commonly used photosensitisers but in the case of HpD or DHE, which are complex and poorly defined mixtures of products with varying fluorescence properties, it is questionable whether the observed fluorescence closely mirrors photoactivity (Moan & Sommer, 1981; Berns *et al.*, 1984). The main aggregates formed with phthalocyanines are dimers whose fluorescence appears to be negligible and whose photoactivity is also very low compared

**Figure 6** Photobleaching effect of red light (675 nm) *in vivo* on bladder 24 h after sensitisation with 5 mg kg$^{-1}$ AlSPc (same as Figure 3a). The sensitiser is progressively degraded with increasing fluence (**a** – after 40 J cm$^{-2}$) and only a thin rim of fluorescence is seen in the mucosa after 80 J cm$^{-2}$ (**b**). If the circulation is arrested before irradiation little loss of fluorescence is seen (**c** – 80 J cm$^{-2}$ *ex vivo*).

to that of monomers (McCubbin, 1985; Spikes & Bommer, 1986). The interpretation of the correlation between fluorescence and photoactivity has been examined previously for AlSPc (Barr *et al.*, 1988; Berg *et al.*, 1989), from which it was concluded that fluorescence detection is selective in that only the photoactive monomers are detected. Therefore these fluorescence estimations reflect the concentration of photoactive sensitiser in tissue which is more relevant than the total amount which will include inactive aggregates.

*Timing of light exposure*

Tissue sensitiser concentration will depend on the time lapse before treatment as well as the administered dose. Again the ideal is not known though most reported series have treated at 48–72 h after sensitisation. Benson's group (1986) treated at 3 h as *in vivo* fluorescence of bladder tumours under UV light was greatest at this time. Not withstanding the difficulties in relating HpD fluorescence to photoactivity, the timing of light delivery should be when the most advantageous ratio of concentration between tumour and normal tissue occurs, which is not necessarily when the highest levels are seen in tumour. Initially after injection there is also a high level in normal tissue especially in well vascularised areas such as the submucosa and serosa. It is only later that the slower clearance of sensitiser from malignant tissue results in a concentration difference. This ratio in rat colonic tumours has been found in our laboratory to be greatest at 48 h, though only about 2:1 (Tralau *et al.*, 1987), and it would not be unreasonable to expect a similar situation in bladder carcinoma. We have shown in this paper that the ratio between normal mucosa and muscle remains fairly constant, at between 3–4:1, though absolute values decline, between 24 h and 72 h so it would therefore seem most appropriate to treat bladder tumours around 48 h after sensitisation. Later than this confers no advantage in the distribution of photosensitiser in normal tissue and it is at 48 h that the most advantageous ratio between tumour and normal tissue may be expected, though this should be clarified when studies of AlSPc uptake in man are possible.

*Photobleaching*

During light exposure some photosensitiser molecules become photodegraded and will neither produce singlet oxygen nor fluoresce. This phenomenon was first described *in vitro* by Moan (1986), and later *in vivo* by Mang *et al.* (1987) who showed a loss of fluorescence and reduction in extractable porphyrin from a mouse mammary tumour. A similar response has been demonstrated in other tumour models. In this work we have demonstrated photobleaching at the microscopic level and shown that this process requires an intact blood supply and has the effect of reducing the amount of photoactive sensitiser in the deeper muscle relative to the superficial layers of the bladder wall. It may be possible to use this phenomenon to increase the selectivity of clinical PDT.

There are two conditions to be satisfied before a PDT effect can occur. Firstly enough singlet oxygen must be produced to cause tissue necrosis. This requires a minimum tissue concentration of photosensitiser as below this threshold level it is destroyed by photobleaching before sufficient singlet oxygen has been produced, whatever the light dose. Secondly a given tissue effect depends on the product of sensitiser concentration and light dose for which there is reciprocity in the ranges most workers have studied (Bown *et al.*, 1986; Barr *et al.*, 1987; Profio & Doiron, 1987). If the sensitiser concentration is increased, the threshold light dose is reduced and vice versa. This reciprocity is lost for low concentrations of photosensitiser near the threshold level (Potter *et al.*, 1987) where photobleaching becomes more important. The therapeutic ideal is to manipulate the difference in concentration of photosensitiser between the urothelium and underlying muscle to achieve photoactive conditions in the former but sub-threshold concentrations in the muscle. In this situation photobleaching of the sensitiser in the muscle layers will spare it from unwanted damage during light exposure though still produce tissue necrosis in the mucosa. Additional selectivity in clinical practice may result from the small differential in photosensitiser concentration to be expected between bladder tumour and adjacent normal mucosa.

In conclusion this fluorescence technique defines and quantifies photosensitiser distribution in tissue on the microscopic level. We have studied AlSPc, but with appropriate wavelength adjustment, it would be equally applicable to the porphyrins. High magnifications may be used to localise fluorescence to cellular structures as sensitivity is much greater than with conventional fluorescence microscopy. Unwanted photobleaching during acquisition of the image is not a problem because of the low excitation powers used, therefore rates of photobleaching of low sensitiser concentrations caused by *in vivo* irradiation can be accurately assessed (unpublished data). We found that a fluorescence intensity gradient of 3–4:1 between superficial and deep layers of the normal bladder wall was established from 24 h sensitisation due to slower elimination from the superficial layers, and it is suggested that the optimum time to treat bladder tumours is around 48 h. If low photosensitiser doses, and necessarily higher light doses, are employed then photobleaching may further improve the selectivity of PDT damage between the superficial and deep layers of the bladder wall. We have since applied these parameters to an animal model of bladder function, and produced a reliable necrosis of the bladder mucosa without damaging the underlying musculature (Pope & Bown, 1991). This has healed by the regeneration of normal tissue without the permanent reduction in bladder capacity or compliance which has been seen in some clinical series subsequent to muscle layer fibrosis.

This work was carried out at the National Medical Laser Centre in the Department of Surgery at University College Hospital, London. A.J. Pope is supported by a research fellowship from the Sir Jules Thorn Charitable Trust. S.G. Bown is supported by the Imperial Cancer Research Fund, whose histopathology services are also gratefully acknowledged. A.J. MacRobert and D. Phillips acknowledge generous support from the Imperial Cancer Research Fund and the Waldberg Trust, S.G. Bown is also supported by the Special Medical Development on Lasers from the Department of Health.

### References

BARR, H., TRALAU, C.J., MACROBERT, A.J. & 4 others (1987). Photodynamic therapy in the normal rat colon with phthalocyanine sensitisationl. *Br. J. Cancer*, **56**, 111.

BARR, H., TRALAU, C.J., MACROBERT, A.J., MORRISON, I., PHILLIPS, D. & BOWN, S.G. (1988). Fluorescence photometric techniques for determination of microscopic tissue distribution of phthalocyanine photosensitisers for photodynamic therapy. *Lasers Med. Sci.*, **3**, 81.

BENSON, R.C. (1986). Integral photoradiation therapy of multifocal bladder tumors. *Eur. Urol.*, **12**, suppl. 1, 47.

BERNS, M.W., HAMMER-WILSON, M., WALTER, R.J. & 4 others (1984). Update and localization of HPD and 'active fraction' in tissue culture and in serially biopsied human tumors. In Doiron, D.R. Gomer, C.J. (eds) Porphyoin localization and treatment of tumors. Alan Liss, New York. (*Prog. Clin. Biol. Res.* **170**, 501).

BERG, K., BOMMER, J.C. & MOAN, J. (1989). Evaluation of sulfonated aluminium phthalocyanines for use in photochemotherapy. A study on the relative efficiencies of photoinactivation. *Photochem. Photobiol.*, **49**, 587.

BOWN, S.G., TRALAU, C.J., SMITH, P.D., AKDEMIR, D. & WEIMAN, T.J. (1986). Photodynamic therapy with porphyrin and phthalocyanine sensitisation: quantitative studies in normal rat liver. *Br. J. Cancer*, **54**, 43.

HARTY, J.I., AMIN, M., WIEMAN, T.J., TSENG, M.T., ACKERMAN, D. & BROGHAMER, W. (1989). Complications of whole bladder dihematoporphyrin ether photodynamic therapy. *J. Urol.*, **141**, 1341.

MANG, T.S., DOUGHERTY, T.J., POTTER, W.R., BOYLE, D.G., SOMMER, S. & MOAN, J. (1987). Photobleaching of porphyrin used in photodynamic therapy and implications for therapy. *Photochem. Photobiol.*, **45**, 501.

MOAN, J.C. (1986). Effect of bleaching of porphyrin sensitizers during photodynamic therapy. *Cancer Lett.*, **33**, 45.

MOAN, J. & SOMMER, S. (1981). Fluorescence and absorption properties of the components of hematoporphyrin derivative. *Photochem. Photobiol.*, **3**, 93.

MCCUBBIN, I. (1985). *The Photochemistry of Some Water-Soluble Phthalocyanines*. University of London, PhD Thesis.

NSEYO, U.O., DOUGHERTY, T.J. & SULLIVAN, L. (1987). Photodynamic therapy in the management of resistant lower urinary tract carcinoma. *Cancer*, **60**, 3113.

POPE, A.J. & BOWN, S.G. (1991). The morphological and functional changes in rat bladder following photodynamic therapy with phthalocyanine photosensitization. *J. Urol.*, **145**, 1064.

POTTER, W.R., MANG, T.S. & DOUGHERTY, T.J. (1987). The theory of photodynamic therapy dosimetry: consequences of photodestruction of sensitizer. *Photochem. Photobiol.*, **46**, 97.

PROFIO, A.E. & DOIRON, D.R. (1987). Dose measurements in photodynamic therapy of cancer. *Lasers Surg. Med.*, **7**, 1.

SPIKES, J.D. & BOMMER, J.C. (1986). Zinc tetrasulphophthalocyanine as a photodynamic sensitizer for biomolecules. *Int. J. Radiat. Biol.*, **50**, 41.

TRALAU, C.J., BARR, H., SANDEMAN, D.R., BARTON, T., LEWIN, M.R. & BOWN, S.G. (1987). Aluminium sulfonated phthalocyanine distribution in rodent tumours of the colon, brain and pancreas. *Photochem. Photobiol.*, **46**, 777.